# Site-Specific Microparticle Inhalation Therapy: A New Approach to Nasopharyngeal Symptoms

**DOI:** 10.3390/ph18091393

**Published:** 2025-09-17

**Authors:** Eride Quarta, Fabio Sonvico, Ignazio La Mantia, Antonio Varricchio, Lucia Gloria, Massimiliano Minale, Niccola Funel, Francesca Buttini, Attilio Varricchio

**Affiliations:** 1 Department of Food and Drug, University of Parma, Parco Area delle Scienze 27/A, 43124 Parma, Italy; eride.quarta@unipr.it (E.Q.); fabio.sonvico@unipr.it (F.S.); 2 Dipartimento di Scienze Mediche, Chirurgiche e Tecnologie Avanzate “G.F. Ingrassia”, Otorinolaringoiatria Azienda Ospedaliero Universitaria Policlinico P.O. “G. Rodolico”Università degli Studi di Catania, 95123 Catania, Italy; igolama@gmail.com; 3 Università Vita-Salute San Raffaele, 20132 Milano, Italy; antovarricchio2001@gmail.com; 4 Dipartimento di Medicina e Scienze della Salute ‘V. Tiberio’, Università del Molise, 86100 Campobasso, Italy; lugloria98@gmail.com (L.G.); attilio.varricchio@unimol.it (A.V.); 5 Indipendent Researcher, Via R.De Blasio, Marigliano, 80034 Napoli, Italy; maxminale@hotmail.com; 6 Section of Laboratory Analysis, Division of Immunohematology, Department of Laboratory Diagnostics, Azienda Ospedaliera USL Nordovest, Via Lippi Francesconi, 55100 Lucca, Italy

**Keywords:** inhalation therapy, microparticles, medical device, low-angle laser light scattering, nasopharyngeal

## Abstract

**Background/Objectives:** Inhalable Microparticles (IMPs) are part of a currently invading field of medicine. In fact, the anatomical district of Rhinopharynx represents a bed for many different pathologies and infections, where the dimension of drug aerosol Microparticles (MPs) represents a discriminating factor to success therapy. The aims of the present work are to demonstrate the efficacy of a new device and its aerosol reproducibility in the nebulization of suspensions to be deposited in the retropharynx. **Materials and Methods:** The Low-Angle Laser Light Scattering (LALLS) method was used to evaluate both the dimension and distribution of MPs. Six different APIs, used usually in Rhinopharynx pathology, were compared in order to investigate the dimension of MP emissions using four different devices. The results of a retrospective study including 74 subjects treated with standard therapy (ST) and the inhalation of nebulized Sobrerol (NS) were performed. Data regarding the persistence of clinical symptoms (i.e., cough and nasal constipation) were acquired. **Results:** No significant statistical differences among all the products tested (*p* > 0.05) were found. One device, Rinubes, demonstrated efficacy and robustness in the fine nebulization of all the pharmaceutical products analyzed. Rinubes delivered an aerosol cloud with significantly lower MMD (66.3 µm) than Mad Nasal and Spray-sol (142.1 and 116.0 µm, respectively), which would allow a higher fraction of drugs to be deposited in the retropharynx. The retrospective clinical study revealed that NS treatment showed higher odds of cough resolution (OR 9.18; *p* < 0.001) with respect to control ST and showed higher odds of nasal symptom resolution (OR 6.7; *p* = 0.043). **Conclusions:** Improved techniques for the administration of inhalable MPs (INPAD) represent significant progress in overcoming the biological and the anatomical barriers in controlling drug release at a specific site. The challenges of nasopharyngeal pathologies offer promising opportunities for the development of non-invasive drug delivery.

## 1. Introduction

Respiratory tract disorders, including asthma, chronic obstructive pulmonary disease (COPD), and rhinitis due to infections, are a significant health problem worldwide. In 2019, it was estimated that a total of 450 million people suffered from chronic respiratory diseases worldwide, causing 4 million deaths, making them the third leading cause of death [1].

The most important diseases affecting the upper airways are rhinitis, rhinosinusitis, otitis, and nasopharyngitis [2,3,4,5]. Acute nasopharyngitis may also include tonsillitis (pharyngotonsillitis) and adenoiditis. These diseases are commonly acute, and the cause is infectious, starting in the nose. Namely, the common cold includes nasopharynx involvement [6]. All these upper airway diseases have common or similar symptoms. Specifically, mucus hypersecretion and defective mucociliary clearance cause mucus accumulation, interfering with airflow passage [7]. Furthermore, chronic inflammation causes turbinate hypertrophy, leading to nasal obstruction [8]. Therefore, the persistence of mucus promotes extraordinary microbial growth, favoring the spread of infection to the paranasal sinuses and middle ear [9]. Posterior rhinorrhea that drips into the pharynx and larynx becomes the trigger for cough; this phenomenon is called postnasal drip and is the onset and cause of rhino-sinus-bronchial syndrome [10]. In particular, nasal function is impaired and significantly affects the lower airways, particularly influencing asthma and sleep [3,5,11]. Thus, the compromised nasal function significantly affects the lower airways [11]. Consequently, a non-patent and non-clean nose promotes, maintains and amplifies respiratory infections and inflammatory diseases [12]. Consequently, an ideal therapy should “open and clean” the nose to ensure the health of the respiratory tract [12]. Therefore, opening and cleaning the nose is the simplest and most useful remedy that can be pursued in daily practice and at all ages. However, the use of clinical treatments is indispensable. The various options include physical, medical and surgical treatments that can control upper airway diseases. Intranasal administration of products and active ingredients represents the safest and most effective way to quickly obtain normal nasal patency, regulate inflammation, fight pathogens and consequently reduce and alleviate nasopharyngeal symptoms [5]. On the opposite side, conventional therapies remain effective up to a certain point and often show limiting aspects, such as systemic side effects [1,13], poor bioavailability of the active ingredient [5,14] and non-optimal pharmacological targeting [5,15]. Furthermore, these therapies often do not attain the specific anatomical site [16,17].

Typically, the methods of administering topical nasal treatments can be divided into three main groups, which include irrigation, nebulization and instillation of drops [18]. Specifically, the nebulization technique and the nebulizer medical device appear to have been originally developed to produce micrometric particles sized 2–10 µm, which can deposit within the nasal cavities. More recently, there has been growing interest in the nasal delivery of nanoparticles (NPs) and in identifying systems capable of generating these particles [16,17].

Inhaled Nanoparticle Therapy (INPT) is a very actual, but also highly debated topic [17]. Recent technological developments have revolutionized the field of NPs by allowing them to be customized in size in order to reach the specific anatomical site for the treatment of the pathology, while avoiding non-specific treatment [19,20,21]. Although NPs conceived as drug carriers have already been successful in the treatment of upper and lower airways [14,19,20,22,23,24,25], the production of other submicrometric particles (i.e., <1 µm) as well as Inhalable Microparticles (IMPs) is part of a currently growing field of medicine [15,26,27,28]. In fact, the anatomical district of the rhinopharynx represents a bed for many different pathologies and infections, where the dimension of drug aerosol microparticles (MP) represents a discriminating factor for successful therapy [14,29,30,31,32,33,34]. Nasal nebulizers are devices designed to deliver microparticles into the upper airways, showing significant potential to improve airway symptoms [24]. Current research focuses on the development of advanced formulations of microparticles or droplets containing nanoparticles for intranasal use, with the aim of overcoming biological barriers and improving drug bioavailability [22,35]. NPs, such as chitosan-based nanoparticles, preserve the active principles from degradation, promote their release and absorption, improving the efficacy of vaccines and drugs [23,25]. Furthermore, methodologies have been developed to evaluate the efficiency of NPs delivery by nebulizers, providing an important tool for the evaluation of micro/nanoflow systems in drug delivery [36]. However, specific evidence seems to confirm that few options are available in order to combine nebulizers producing IMPs of drug production [26,37].

This study aims to develop the following three main work packages to select and test the best aerosol production for inhalation therapy: (1) demonstrate the performance of four devices (nebulizers and Medical Devices—MDs) in producing nebulized liquid suspensions and their reproducibility; (2) select one device, selecting it based on the target particle size for deposition in the retropharynx; (3) support points 1 and 2 of the study by presenting data from a human study conducted using the selected device.

## 2. Results

### 2.1. Particle Size Distribution of Aerosol Produced by Saline Solution with Four Different Nasal Devices

In order to compare the four different nebulizing devices, saline solution was aerosolized using different nasal insufflators.

Three of them were constituted of a rubber nebulizer head attached to a syringe. They differed in the size and number of holes nebulizing the liquid. In parallel, a nasal douche was tested since it is considered a mature and well-established technology.

**Figure 1:**
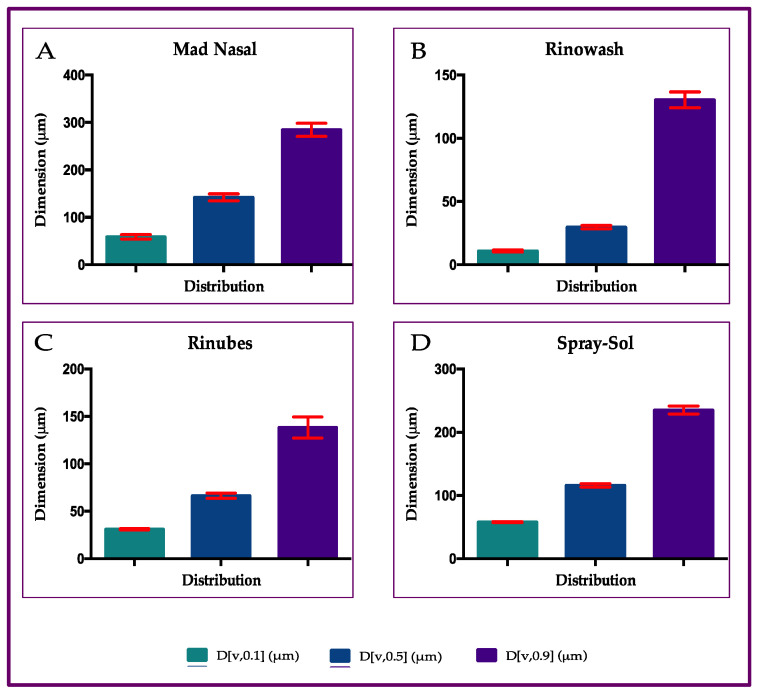
Dimension of aerosol particles of saline solution from four different devices: (**A**) Mad Nasal; (**B**) Rinowash; (**C**) Rinubes; (**D**) Spay-sol.

The devices provided different performances in the droplet size emitted. In total, 90% of the micro particles had a volume diameter of less than 284.8 µm when emitted from MadNasal, 130 µm from the Rinowash, 138.4 µm for those emitted from Rinubes, and 235.1 µm for those emitted from Spray-sol. The ANOVA test revealed that among all four devices, there are statistical differences (*p* < 0.001) concerning the production of microparticles and the parameter D[v,0.50] (Figure 1).

**Figure 2:**
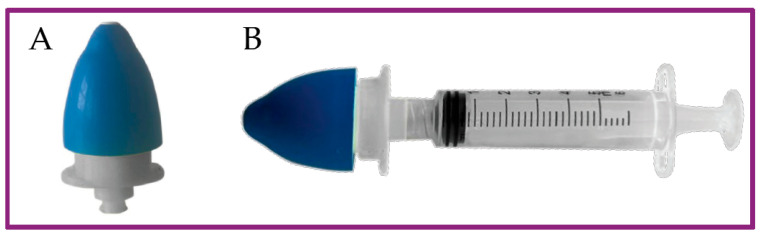
Representation of Rinubes. The Rinubes device has been meticulously designed to deliver a gentle and safe aerosol, ensuring maximum comfort for children’s delicate nasal passages. The distinctive aspect of this innovation lies in the precision of the aerosol cloud generated: the droplets have a uniform size, within a recognized range, to improve the product’s performance and efficacy. (**A**) Rinubes device; (**B**) configuration of Rinubes: association with the screw syringe for the administration of the pharmaceutical products for the treatment. Manufactured in Italy.

**Figure 3:**
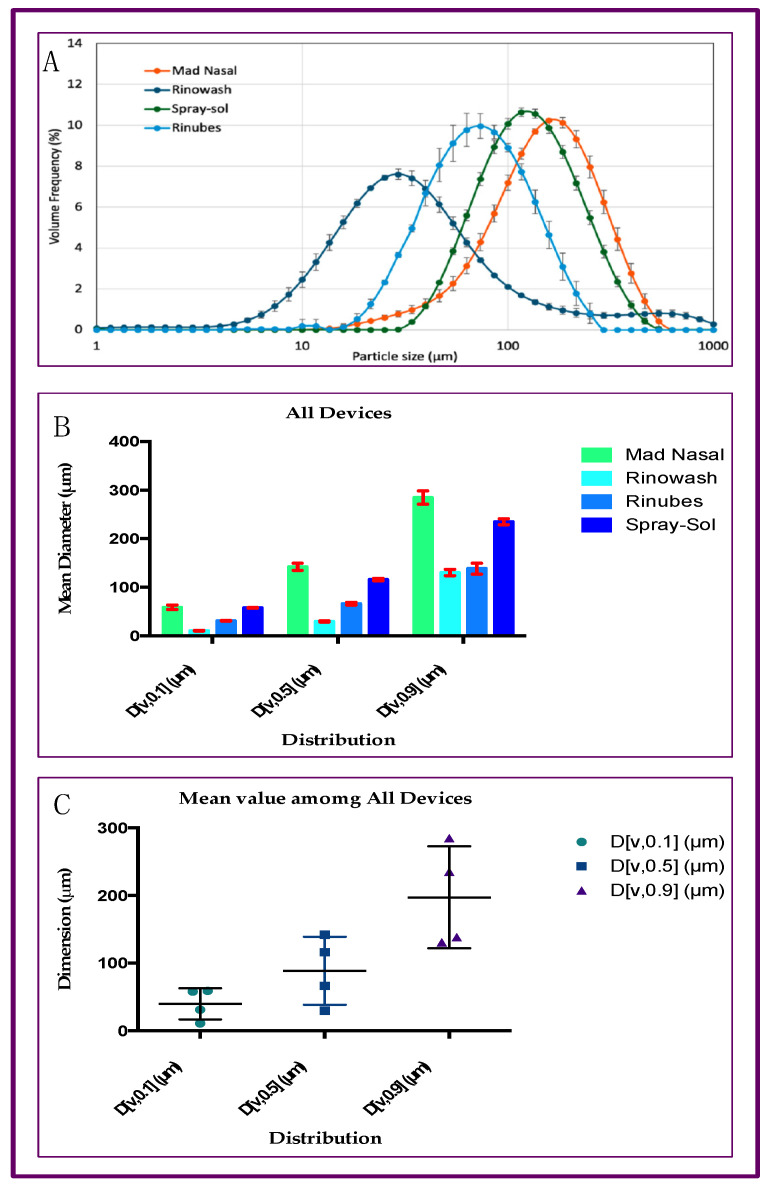
Cumulative analyses concerning the four devices tested: (**A**) Plotter concerning dimension and frequency of particles; (**B**) ANOVA analyses of distribution.; (**C**) mean value analyses among tested medical devices; statistical difference was observed among the products (*p* < 0.001) and the distribution of micro particles (*p* < 0.001).

The plume-generated saline solution tested with Mad Nasal and Spray-sol showed an MMD of 142.1 µm and 116.0 µm, respectively. On the contrary, Rinubes (Figure 2) produced a finer plume with an MMD of 66.1 µm. Rinowash, which has a different mechanism of aerosol production, revealed the lowest value of MMD (i.e., 30 µm: Figure 3). The dimensions of aerosol particles of saline solution from four different devices are illustrated in Figure 3.

Rinowash is a nasal irrigation product that has to be connected to a traditional pneumatic compressor for aerosol therapy, generating an air flow of 15–20 L/min and it allows a complete treatment of the upper airways in 1/3 min. It is reported that it generates particles with a diameter greater than 10 microns, it acts exclusively at the level of the upper airways and nebulizes 5 mL of solution in one minute. The generation of finer particles by this nasal shower is due to the more efficient thrust that the compressor imparts to the liquid compared to the force that is generated manually using a syringe with the nebulizer attached. On the other hand, this system is more sophisticated, requires electricity and a more detailed understanding of the procedure for using and cleaning the device.

Finally, for all the manually activated devices (Mad Nasal, Spray-sol and Rinubes), there was not a fraction of particles less than 10 microns. Hence, the data show that all the aerosols generated are of a size not suitable to be inhaled and reach the lungs. The plume produced is specifically useful for the treatment of the upper airways. Thus, the aerosol size distribution was demonstrated to be suitable for nasal deposition, avoiding the penetration of droplets into the lungs. Rinowash, producing a finer cloud, generated about 9.5% of droplets < 10 µm, potentially capable of reaching the lungs. It was specified in the introduction that the particles must be fine enough to reach the nasopharynx. For this reason, we analyzed the portion of generated droplets between 10 and 30 µm because these are potentially more effective for the therapy. In this sense, the measured fraction corresponded to 0.34 and 2.14% for Spray-sol and Mad Nasal, respectively, while it reached 7.18% for Rinubes, indicating a more efficient generation of fine particles for this device. In line with what emerged from the MMD data, Rinowash showed a consistent fraction below 30 µm and equal to 51.6%. Among the manually activated devices, Rinubes showed the lowest MMD values, indicating a higher probability of droplets depositing in the rhino pharynx portion. At the same time, since no particles were generated with a size < 10 µm, the risk of particles directed to the lungs is minimised.

The global mean for all three parameters reported in Figure 1 revealed that Rinubes achieved a consistent production of droplets, as the D[v,0.50] differences among the compounds used were not statistically significant (ANOVA test; *p* > 0.05). In contrast, the differences between the undersized D[v,0.10] and oversized D[v,0.90] particles were statistically significant among products (ANOVA test; *p* < 0.0001). These data suggest that Rinubes (Figure 2) produced a high number of particles with a size suitable for the treatment of the nasopharyngeal site (Figure 3).

### 2.2. Particle Size Distribution of Aerosol Produced by Six Different Drugs, with Rinubes Device

**Figure 4:**
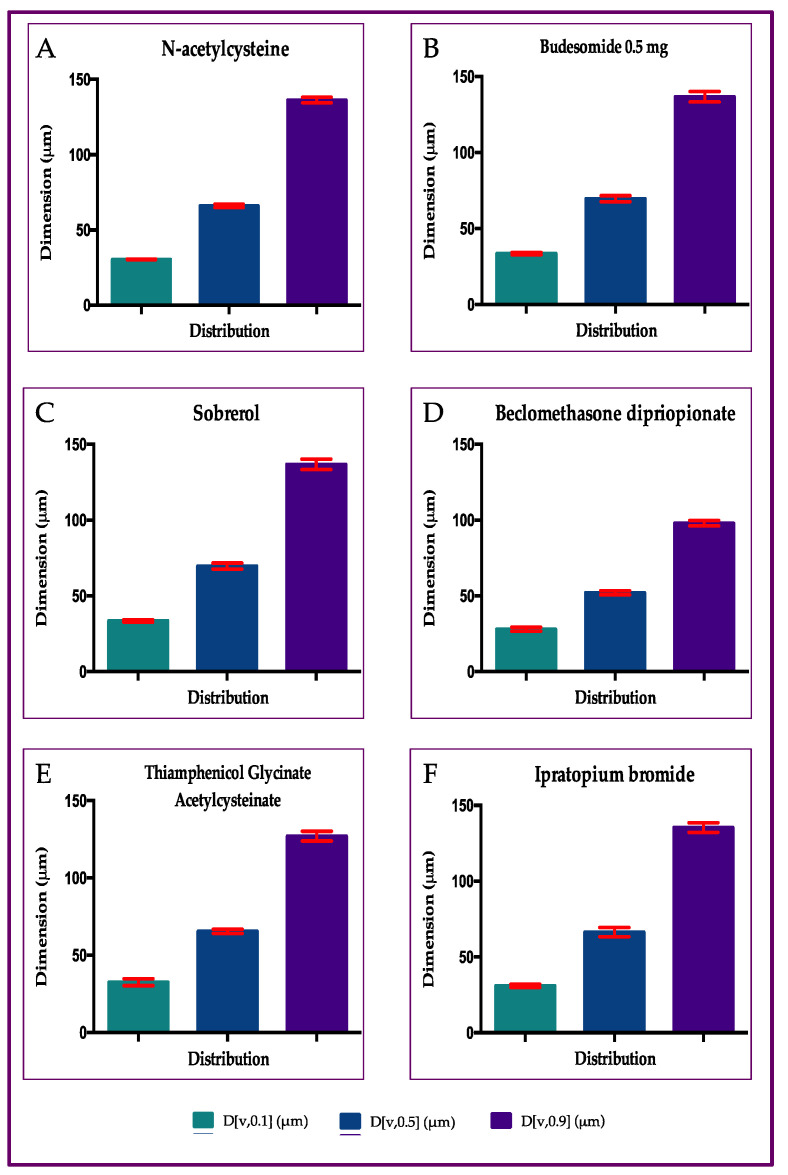
Analyses of dimension of particles from aerosol from Rinubes device: (**A**) N-acetylcysteine; (**B**) Budesonide 0.5 mg; (**C**) Sobrerol; (**D**) Beclomethasone dipropionate; (**E**) Thiamphenicol glycinate acetylcysteinate; (**F**) Ipratropium bromide.

**Figure 5:**
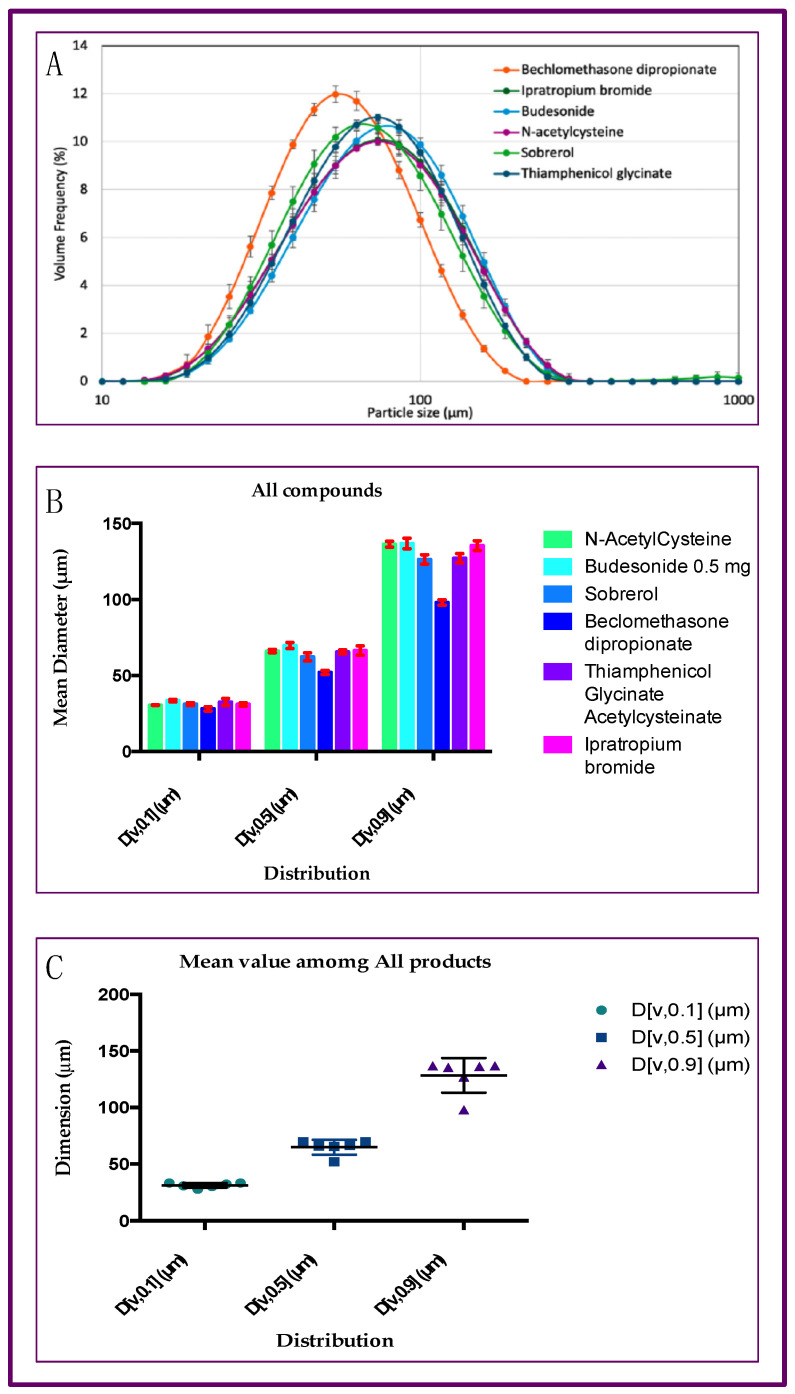
Cumulative analyses concerning the six API tested: (**A**) average and standard deviation results of size distribution for the six tested drugs with Rinubes; (**B**) ANOVA analyses of particle size distribution (PSD). (**C**) Graph plot representing the mean particle distribution among all products. No statistical difference was observed among the products (*p* > 0.05, while statistical difference was observed among the dimensions of size distribution.

The results presented in Figure 4 show the median mass diameter (MMD) at D[v,0.5] and the percentiles undersize D[v,0.1] and oversized D[v,0.9] of the aerosol particles produced by nebulizing six formulations (six commercial liquid medicinal products) using four different medical devices, i.e., nebulizers. The analysis of particles indicated the values of diameter corresponding, respectively, to 10% and 90% of the droplet population. No significant statistical differences among all products tested (*p* > 0.05) were evidenced, comparing the results concerning the following values: D[v,0.1], D[v,0.5] and D[v,0.9] (Figure 4). In total, 90% of droplets generated by the Rinubes insufflator had a volume diameter of less than 137 µm. All drugs tested with Rinubes showed an MMD between 52.1 and 69.7 µm. The lowest MMD value of dimension was reported by beclomethasone dipropionate (52.1 µm; Figure 4 and Figure 5). Rinubes demonstrated efficacy and robustness in fine nebulization of all pharmaceutical products analyzed, indicating that the device is efficient with all types of formulation tested, whether they are cortisone suspensions or solutions with different dosage strengths and different compositions of excipients. The medical device showed a good reproducibility since the D[v,0.5] RSD was <3% and the D[v,0.1] and D[v,0.9] RSD were <5%.

### 2.3. Results of Patients Treated with ST and NS

**Figure 6:**
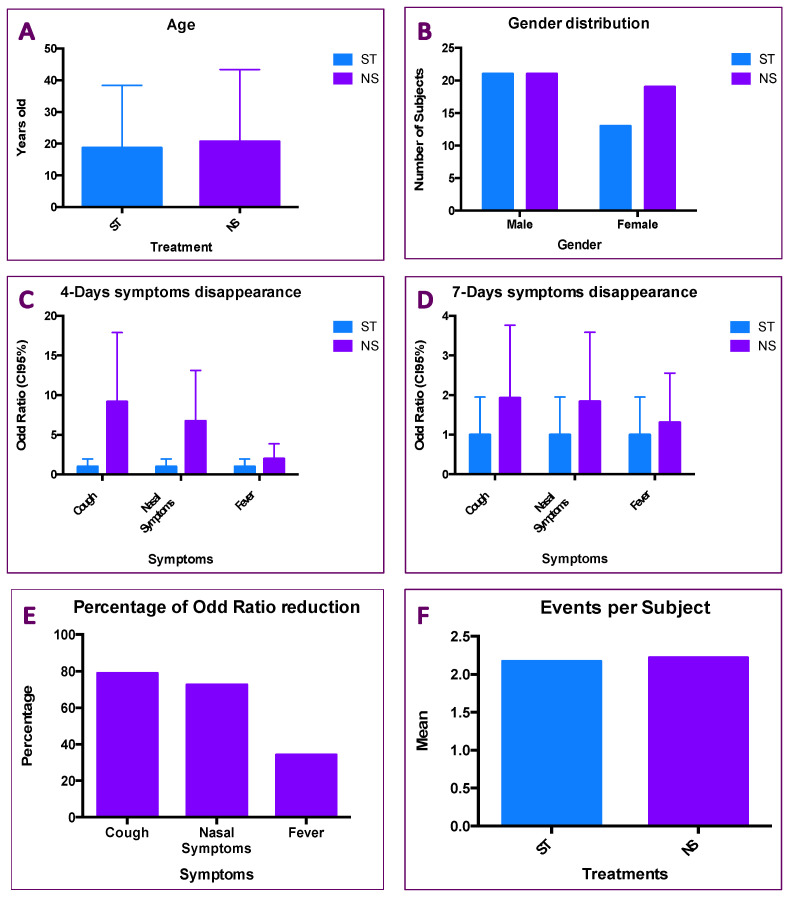
Characteristics and analyses of cohort of patients. Among the subgroup previously published the focused analyses included 74 out of subjects (41.80%) of treated patients. Thirty-four of them were treated with Standard Treatment (ST), while forty subjects were treated with Nebulizer Sobrerol (NS). (**A**) Age distribution: ST = Standard Therapy; NS = Nebulizer Sobrerol. No significant statistical differences were found by The Student’s Test application (*p* = 0.6857). (**B**) Gender distribution: ST = Standard Therapy; NS = Nebulizer Sobrerol; No significant statistical differences were found by Chi-square Test application (*p* = 0.4847). (**C**) Disappeared symptoms after 4 days: ST = Standard Therapy; NS = Nebulizer Sobrerol; Significant statistical differences were found by ANOVA Test application, concerning the differences among the treatments (*p* < 0.0001) and the differences among the symptoms (*p* < 0.001) in NS. (**D**) Disappeared symptoms after 7 days: ST = Standard Therapy; NS = Nebulizer Sobrerol. Significant statistical differences were found by ANOVA Test application concerning the differences comparing the treatments (*p* < 0.0001), while no statistical differences were found comparing the symptoms (*p* < 0.3302) in NS. (**E**) Percentage of Odd Ratio (OR) reduction in symptoms (in percentage). The difference of Odd Ratio (OR; CI 95%) expressed in percentage was calculated as (OR4Days-OR7Day)/OR 4Days. Greater differences were evaluated for the cough and nasal symptoms, while the fever symptom reported lowest percentage reduction. (**F**) Number of infection recurrence (IR): Number of mean IR for subject; ST = Standard Therapy; NS = Nebulizer. No significant statistical differences (Ns) were found using the Student’s Test application (*p* = Ns).

The two subgroups mentioned above, investigated in this retrospective study (ST vs. NS), showed a similar distribution concerning the mean age and the gender distribution. In particular, in the ST group, the mean age was equal to 18.70 ± 3.377, while the NS showed a mean age equal to 20.70 ± 3.377 (Figure 6A). Nonetheless, the gender distribution revealed an equal distribution of a M/F ratio among the treatments. Inside the ST group there were 21 males out of 34 subjects, indeed in the NS group there were 21 males out of 40 subjects (Figure 6B). The unpaired Student’s test (Figure 6A and the Fisher’s test (Figure 6B) revealed no statistical differences among the treatments (Figure 5A; *p* = 0.6857 and Figure 5B; *p* = 0.4847).

The ST group was considered the reference group, then the Odds Ratio (OR) concerning three parameters (i.e., cough, nasal symptoms and fever) was calculated. The ORs for symptom disappearance at day 4 were 9.18, 6.73 and 1.99 for the cough, nasal symptoms and fever, respectively (Figure 6C; *p* < 0.0001). The ORs for symptom disappearance at day 7 were 1.93, 1.94 and 1.31 (Figure 6D; *p* < 0.0001), for the cough, nasal symptoms and fever, respectively. Comparing the ORs in the NS group, a significant reduction of them was observed for the cough (−78.98%), for the nasal symptoms (−72.66%), but not for the fever (−34.18%; Figure 6E). The results indicate that NS therapy effectively reduces cough and nasal symptoms as early as day four, with a significantly higher OR compared to the control treatment. The NS treatment also shows up to two times greater efficacy on cough symptoms compared to the control treatment four days after starting treatment (Figure 6C). By day seven, the OR values decrease by up to 78% (Figure 6D) but remain in line with the OR observed for fever and still maintain a high ratio compared to the control treatment (Figure 6E). Finally, no difference in terms of symptoms per patient was observed (ST = 2.17; NS = 2.22). The data of NS treatment seem to indicate that the treatment affects the three symptoms investigated in an independent manner. No significant association among the age, gender or events per patient (Figure 6F) was found.

## 3. Discussion

Rhinosinusitis is a widespread disease worldwide, resulting in significant costs to society in terms of healthcare costs and lost productivity due to work absence [13]. Acute rhinosinusitis (ARS) has an annual prevalence of 6–15% and is usually the consequence of a viral common cold. ARS is usually a self-limiting disease, but serious complications have been described, leading to life-threatening situations and even death [38]. It is one of the most common causes of antibiotic prescription, and proper management is extremely important in the context of the global antibiotic resistance crisis. Chronic rhinosinusitis (CRS) is a significant health problem, affecting 5–12% of the general population [39]. All conditions affecting the upper airways (i.e., rhinitis, rhinosinusitis, otitis, and nasopharyngitis) are dangerous to health [2,3,4,5]. So far, acute nasopharyngitis may also include tonsillitis (pharyngotonsillitis) and adenoiditis. All these conditions present themselves in an acute trait. Indeed, they are located as nasopharynx involvements [6]. Symptoms of ARS or similar conditions often last a few days, but no more than a week, but the cough persists longer and can often lead to a reduction in quality of life [40]. In this regard, a cough can be associated with two opposing situations: excessive mucus production or reduced mucus secretion. These two conditions essentially translate into a productive cough and a dry cough. Both types of cough require appropriate treatment. Mucus does indeed have protective and beneficial effects, but if overproduced, it has harmful effects [41]. Therefore, a goal in managing patients with a cough is to ensure adequate mucus production, meaning that it is neither excessive nor insufficient [42]. In this regard, Sobrerol is a mucoactive agent with additional activities that may be beneficial to patients with ARS. Current clinical experience in primary care has shown some interesting results. Pharmacological, medical, and surgical treatments can certainly treat upper airway diseases (ARDs). Intranasal administration of active ingredients represents the fastest, safest, and most effective method to rapidly restore normal nasal space, reduce inflammation, fight pathogens, and ultimately alleviate symptoms [5]. Precisely, because of this ease of immediate treatment from the outside in, not only have new pharmacological applications been developed [25,27], but the methods of administration have also grown enormously [17,20,23,24,35]. Focusing on this point carefully, inhaled nanoparticle therapy (INPT) is a very current but also much-debated topic [22]. However, it should be noted that confusion can arise when talking about inhaled particles based on their size [5,19,27,31]. It is important to underline for this reason that size makes a difference in reaching the target site for treatment [4,5]. For this reason, this work took into consideration the size of the particles in relation to the specific treatment site. Our results highlight that particle size is important for effectively treating ARS symptoms. Specifically, we compared four devices that demonstrated efficacy in producing the micron-sized particles needed for the specific treatment of these upper respiratory tract conditions [14,34,35]. Particle size must not be too small to enter the lungs [19,30] or too large to effectively reach the retropharynx [11]. In particular, one of the devices used (i.e., Rinubes) was shown to produce micrometric particles of the appropriate diameter. In particular, one of the devices used (i.e., Rinubes) produced micrometric particles of the appropriate diameter. The size of these particles produced by the Rinubes medical device is consistent with those used for pharmacological treatment through the nasal cavities [22]. Nonetheless, the above-mentioned pathological conditions are often accompanied by mucus overproduction or reduced mucus secretion. These two manifestations are two different situations: a productive cough and a dry cough, respectively. Both types of coughs require appropriate treatment. Mucus does indeed have protective and beneficial effects, but if overproduced, it has harmful effects [43]. Therefore, a goal in managing patients with a cough is to ensure adequate mucus production, meaning that it is neither excessive nor insufficient [42]. In this regard, Sobrerol is a muco-active agent [43,44], with additional activities that may be beneficial for patients with upper respiratory tract infections [45]. The results reported in Figure 6 showed the bigger effect of the nebulizer Sobrerol (NS) compared to the standard treatment (ST). Nevertheless, the OR of the NS group (against the cough) showed the highest value in the disappearance of symptoms after 4 days (Figure 6C) and 7 days (Figure 6D). Finally, the two groups of patients were homogeneous in terms of age, gender distribution and number of events per patient. Thus, the results shown appear to be independent of patient demographics.

## 4. Materials and Methods

### 4.1. Medical Devices and Pharmaceutics

In the first phase of the work, particle size distribution of aerosols was assessed for physiological solution 0.9% Sodium Chloride aerosolized by:

- Rinubes, (ADL Farmaceutici, Milan, Italy)
- Mad Nasal, (Sakura, Lonato del Grada (BS), Italy)
- Spray-sol, Buona spa, Sesto Fiorentino (FI), Italy
- Rinowash, (Air Liquide Medical Systems, Milan, Italy)

Indeed, the assessment of particle size distribution by an aerosol of the following six different medicinal products, produced by ADL Farmaceutici, Milan, Italy nasal insufflator (Rinubes). The experiment included the formulation of the following compounds:

- N-acetylcysteine 300 mg/3 mL (Fluimucil^®^, Zambon spa, Milan, Italy)
- Budesonide 0.5 mg/2 mL, (Aircort^®^, Italchimici, Moncalieri (TO), Italy)
- Sobrerol 40 mg/3 mL (Pharm@idea s.r.l., Travagliato, Italy)
- Beclomethasone dipropionate 0.8 mg/2 mL (Clenil Aerosol^®^, Chiesi Farmaceutici spa, Parma, Italy)
- Thiamphenicol glycinate acetylcysteinate 0.5 mg/4 mL (Fluimucil antibiotico^®^, Zambon spa, Milan, Italy)
- Ipratropium bromide 0.5 mg/2 mL (Atem^®^, Chiesi Farmaceutici spa, Parma, Italy)

### 4.2. Patients

This case study was conducted retrospectively by extracting data previously described in the literature [43]. The inclusion criteria included recruiting subjects over 3 years of age, of either sex, with a history of recurrent respiratory infections. Each physician selected the patients they managed by prescribing Sobrerol. Additionally, each physician included a group of patients with upper respiratory tract infections (AURI) treated only with standard treatment for infections. Exclusion criteria included age under 36 months, epilepsy, severe respiratory insufficiency, uncontrolled asthma, or severe physical debilitation. The following data were harvested for each patient: age, gender, treatment, 4-day symptoms reduction, 7-day symptoms reduction and number of recurrences for the patient.

### 4.3. Particle Size Assessments

Measurements were performed with a Malvern Instrument laser diffractometer, Spraytec (Malvern Panalytical, London, UK), validated with Duke Standard Uniform Polymer Microspheres 8.9 µm ± 0.4 (batch #248770, Thermo Scientific, Monza, Italy).

The FDA’s draft guidance recommends the use of laser diffraction to determine the droplet size produced for a given product. Laser diffraction systems, such as Malvern Spraytec, calculate the size of droplets by measuring the intensity of light scattered by particles as a function of angle. The applicable range according to ISO13320:2020 [46] is 0.1–3000 µm. The analysis allows the determination of an aerosol particle size distribution directly by spraying the cloud through the laser beam. D[_v,0.5_] is the Volume Median Diameter (VMD), which refers to the midpoint droplet size, where half of the volume of spray is in droplets smaller than the mean, and half of the volume is in droplets larger than the mean. The percentiles undersize D[_v,0.1_] and D[_v,0.9_], indicating the values of the corresponding diameters were, respectively, 10% and 90% of the droplet population. For the assessment, 3 mL of each drug was introduced into a 5 mL syringe connected to the nasal insufflator. The Rinowash device was filled with 3 mL and connected to a pneumatic aerosol therapy apparatus as reported in the leaflet. A content of a few vials equal to 10 mL was mixed in a beaker and 3 mL withdrawn for each analysis. In detail, the compressor employed was Pic AirFamily Solution (Pikdare, Como, Italy). Three measurements were performed for one insufflator characterization for durations of 5 s. In the case of Rinowash, the sampling period was 30 s in length.

The nasal device loaded with 3 mL of APIs or physiologic solution was positioned on a polystyrene support oriented at a 45° angle and actuated in front of laser. The distance between the orifice of the spray nozzle and the beam path was maintained at 5 cm, while the distance between the spray and the detector window was 10 cm. Obscuration, a measure of the amount of light scattered by the sample, was in the range 11–16%.

All the data were analysed and used for the PSD calculation. Residual value, representing the deviation of the diffraction profile measured from the theoretical model, remained lower than 1% for a good correlation between the experimental data and the model applied for data analysis. Mie theory was employed for the data analysis. It describes the scattering of electromagnetic radiation by spherical particles. It is a solution to Maxwell’s equations for scattering by a homogeneous sphere and provides a complete angular distribution of scattered light intensity as a function of particle size, refractive index, and the wavelength of incident light. Sample refractive index was 1.51 and for air was 1.00.

Droplet size varies considerably during a spray event, so the Guidance for Industry—Bioavailability and Bioequivalence Studies for Nasal Aerosols and Nasal Sprays for Local Action (2 April 2003) recommends the use of data from the fully developed phase to ensure statistically valid comparisons between different products. The fully developed phase can be defined in the data handling software, based on a time window in which all three size parameters (D[_v,0.1_], D[_v,0.5_] and D[_v,0.9_]) are stable (plateau of the measurement). Hence, for this reason, the measurements in this work were performed considering all the cloud profiles produced by the 3 mL nebulizer.

### 4.4. Treatments

Two groups of patients (comprising a total of 74 subjects) were selected from a larger pool of 177 pharmacologically treated subjects [43]. These two groups included subjects retrospectively collected and managed to intercept most respiratory infections. The data harvesting started immediately in the autumn season and continued throughout the “cold season”. In the control group, the patients performed Standard Therapy (ST), usually including antibiotics, antipyretics, and anti-inflammatory drugs (ST; 34 subjects), while the second group was treated with Nebulized Sobrerol (NS; 40 subjects). The nebulization was performed by Rinubes. All treatments were performed with the same device, and both groups followed the same treatment regimen (two applications per day for three days). The data concerning the most important physiological parameters (i.e., cough, nasal symptoms and fever) were recorded after four and seven days from the beginning of treatment.

### 4.5. Statistical Analyses

The software (GraphPad 8.0 version) for Apple Computer was used for statistical analysis (PRISM, San Diego, CA, USA). All the parameters measured in this study were evaluated by using the classical descriptive statistics of mean, SD, minimum and maximum (for quantitative variables), and frequencies (for qualitative variables). All statistical results were considered significant if the *p*-value was less than 0.05 (*p* < 0.05). The Shapiro–Wilk test was performed in order to determine whether the data were parametrically distributed. Both W- and *p*-values for the BMI data were calculated (W = 0.980 and *p* = 0.574). These values justified the implementation of parametric tests for the BMI analyses. Statistical analysis of variance (ANOVA) was performed to determine the significance (*p*-value) of particle size data among the curves, concerning the comparison between the product and the devices.

## 5. Conclusions

Improved techniques for the administration of inhalable NPs (INPAD) and especially inhalable microparticle IMPs represent significant progress in overcoming biological and anatomical barriers in controlling drug release at specific sites, such as the nasopharyngeal. The challenges of nasopharyngeal pathologies offer promising opportunities for the development of non-invasive drug delivery. The subcategory of IMPs offers several advantages, including precise targeting of drugs in the nasal pharynx, improved drug absorption and bioavailability, and reduced systemic side effects. The results of patients reported in these analyses seem to validate the combination of the medical device and drug Sobrerol, with respect to the control treatment. The medical device “Rinubes” seems to be able to be used for the diffusion of a wide range of pharmacological agents and to consolidate a controlled and reproducible release of the drug, which is essential for achieving the therapeutic result.

## 6. Patents

1. Utility model no. 202020000003820 improved nasal nebulizer; Italian patent for industrial invention no. 102020000015844—use of salt–bromine–iodine waters for the nasal treatment of inflammation in the three areas of the upper airways and medical devices adapted for such treatment.
2. European patent application for industrial invention no. 21748658.8—use of salt–bromine–iodine waters for the nasal treatment of the inflammation of the three areas of the upper airways and medical devices adapted to such a treatment.

## Data Availability

All data included in this study will be made available upon request. The data owners are Dr. Eride Quarta, Professors Francesca Buttini and Attilio Varricchio. The original contributions presented in this study are included in the article. Further inquiries can be directed to the corresponding author.
